# Neoadjuvant Hypofractionated Radiotherapy and Chemotherapy in High-Grade Extremity Soft Tissue Sarcomas: Phase 2 Clinical Trial Protocol

**DOI:** 10.2196/resprot.6806

**Published:** 2017-05-25

**Authors:** Ranyell MSB Spencer, Samuel Aguiar Junior, Fabio O Ferreira, Paulo R Stevanato Filho, Bruna EC Kupper, Maria LG Silva, Celso A Mello, Tiago S Bezerra, Ademar Lopes

**Affiliations:** ^1^ Sarcoma and Colorectal Tumors Pelvic Surgery Department AC Camargo Cancer Center São Paulo Brazil; ^2^ Sarcoma and Colorectal Tumors Radiation Therapy Department AC Camargo Cancer Center São Paulo Brazil; ^3^ Sarcoma and Colorectal Tumors Clinical Oncology Department AC Camargo Cancer Center São Paulo Brazil

**Keywords:** soft tissue sarcoma, neoadjuvant treatment, hypofractionated doses, radiotherapy, dose fractionation, toxicity

## Abstract

**Background:**

Neoadjuvant radiotherapy (RT) and chemotherapy are applied to large, high-grade extremity soft tissue sarcomas to treat metastatic disease earlier and sterilize margins to perform R0 surgery. However, preoperative RT increases wound complication rates (rWC), delaying adjuvant chemotherapy or preventing it from being administered altogether. Hypofractionated neoadjuvant RT can be offered in this situation, concomitant to chemotherapy, allowing patients to receive chemotherapy as a preoperative treatment in less time with an acceptable rWC.

**Objective:**

The objectives of this protocol are to maintain low rates of morbidity and mortality compared to literature data, improving survival rates and avoiding poor responders from receiving unnecessary adjuvant chemotherapy.

**Methods:**

This noncontrolled, single-arm, phase 2 clinical trial recruited patients aged over 18 years with high-grade soft tissue sarcomas in the girdles or extremities. Three neoadjuvant chemotherapy (ifosfamide and doxorubicin) cycles were administered with 5 days of hypofractionated RT (25 Gy in 5 fractions) in the second cycle of doxorubicin only. Viable cell counts in the surgical specimen were measured, and patients in whom this value was less than 30% continued to receive an additional 3 full chemotherapy cycles as adjuvant treatment.

**Results:**

Primary endpoint will be disease-specific survival measured by the evaluation of local and distant recurrence after neoadjuvant treatment. The secondary endpoints will be wound complication and amputation rates and chemotherapy toxicity. We also will record the viable cell rates after the schema and correlate this with survival.

**Conclusions:**

As seems with other solid tumors, hypofractionated RT has comparable efficacy and safety as conventional fractionation. This modality of treatment combined with chemotherapy could increase the pathological response rates and improve the outcomes of select patients.

**Trial Registration:**

ClinicalTrials.gov NCT02812654; https://clinicaltrials.gov/ct2/show/NCT02812654 (Archived by WebCite at http://www.webcitation.org/6qC3puBOy)

## Introduction

Soft tissue sarcomas (STSs) are rare neoplasms and account for 1% of all solid tumors in adults. In 2013, there were 11,410 new cases diagnosed in adults and children in the United States, with 4390 expected deaths [1]. There are no data on such estimates in Brazil. By inference, using the American population, we have predicted an annual incidence of 7060 cases for 2013.

Approximately 50% to 60% of cases develop in the limbs, which can differentiate into more than 50 histological subtypes [2]. High-grade sarcomas, which are deep and larger than 5 cm, are locally aggressive and cause distant metastases, primarily to the lungs. The risk of lung metastasis from high-grade tumors ranges from 34% in 5- to 10-cm lesions to 43% for those over 10 cm [3].

The treatment for STSs, which are localized but not amenable to adequate resection, is multimodal in most cases and often involves surgery, chemotherapy, and radiotherapy (RT) [3]. Nevertheless, selecting patients to receive chemotherapy as adjuvant treatment is an option to avoid unnecessary toxic treatment in cases that are considered to be nonresponsive; this approach increases the rates of limb sparing but fails to improve overall survival (OS)—patients continue to die of metastatic disease, especially in the lung. Further, choosing an RT scheme that reduces wound complication rates might increase the number of patients who receive chemotherapy when it is indicated.

To increase the tumor necrosis rate in patients with STSs of the extremities that are not amenable to adequate resection, with regard to their survival, we have proposed a treatment regimen in which neoadjuvant chemotherapy is administered with concomitant hypofractionated RT and adjuvant chemotherapy for select cases, depending on the viable cell count. Our aim is to maintain morbidity and mortality rates compared with the current regimen, improve survival, and spare patients who are considered to be poor responders from unnecessary chemotherapy.

## Methods

### Overview

This prospective, uncontrolled, phase 2 study began in February 2015. Recruitment is expected to be completed by December 2018. A total of 70 patients are to be recruited in this period, based on the demographics of new cases that enter our service per year and the criteria for compatibility with the study. For a rise in disease-free survival from 50% to 70%, we calculate that we will need 70 patients, with an alpha error of .05 and statistical power of 80% (1-tailed test). These parameters are also considered for increasing the response rate from 15% to 20%.

The recruitment will target patients aged 18 to 75 years with nonmetastatic STSs of the extremities with high histological grade, including the pelvic and shoulder girdles, as confirmed by an anatomopathological exam. These patients must have primary or recurrent tumors that are not amenable to resection, with an adequate 3-dimensional margin of 1.0 to 2.0 cm or, in those in whom preservation of the limb is not possible, must not have undergone chemotherapy or prior RT before the clinical trial. The Karnofsky index [4] should be greater than 70%, and the following criteria must be met: leukocyte count >3500/mm^3^, neutrophil value >1500/mm^3^, platelet count >100,000/mm^3^, serum creatinine <1.5 mg/dL, bilirubin serum concentrations within normal limits, transaminase levels up to twice normal values, and no signs of coronary artery disease as determined by ejection fraction.

Patients who have been diagnosed with embryonal rhabdomyosarcoma, primitive neuroectodermal tumors, chondrosarcomas, osteosarcomas, or HIV infection or have refused to sign the informed consent form, as approved by the Research Ethics Committee, will be excluded from the study. Patients with pelvic or thoracic organs in the radiation field are also excluded.

Recent resection was defined as surgery up to 3 months before admission to our service, whereas relapsed tumor was considered a tumor that appeared after 3 months of any prior surgical treatment.

### Toxicity

Treatment will be continued until disease progression (according to Response Evaluation Criteria In Solid Tumors [RECIST], version 1.0) [5], unacceptable toxic effects, withdrawal of consent, or death. Adverse events will be graded per the National Cancer Institute Common Toxicity Criteria for Adverse Events (version 5.0) [6].

All patients will be staged with regard to local disease by magnetic resonance imaging (MRI). Chest computed tomography (CT) will be used to evaluate the presence of distant disease. For patients with liposarcoma of the extremities, CT or ultrasonography of the total abdomen with the pelvis will be performed. All tumors, including recurrent tumors, will be classified per the American Joint Committee on Cancer Cancer Staging Manual, 7^th^ Edition [7].

### Chemotherapy and Radiotherapy

The treatment plan comprises 1 cycle every 21 days for a total of 3 preoperative cycles in conjunction with hypofractionated RT. Doxorubicin 75 mg/m^2^ will be administered intravenously over 30 minutes, day 1, in cycles 1, 2, and 3; ifosfamide 9.0 g/m^2^ will be delivered intravenously over 2 hours, days 1 to 5 in cycles 1 and 3; mesna will be given at 100% of the ifosfamide dose, with half infused 15 minutes prior to and half infused 4 hours after beginning the infusion of ifosfamide; and filgrastim 300 mcg will be administered subcutaneously, days 6 to 10 in cycles 1, 2, and 3.

**Figure 1:**
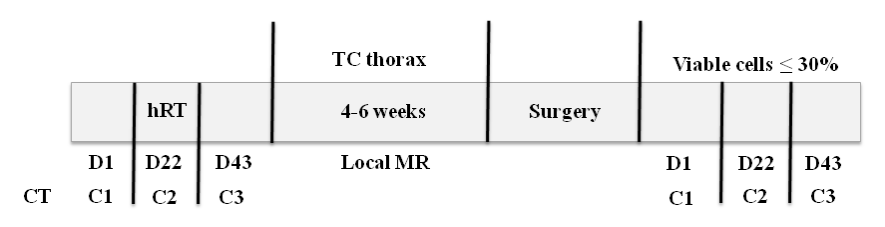
Scheme and treatment schedule illustration.

Surgery will then be performed 4 to 6 weeks after the end of the third cycle. Three additional cycles of chemotherapy, similar to cycle 1, will be offered to cases with ≤30% viable cells in the surgical specimens (thus considered to be good responders). RT (intensity-modulated radiation therapy or 3-dimensional) will be performed with the following doses and fractions: 25 Gy/5 fractions of 500 cGy on consecutive days from Monday to Friday, starting on day 1 of the second chemotherapy cycle (C2) (see Figure 1).

After the phase RT/induction chemotherapy, the patients are examined after 3 weeks of each chemotherapy cycle by the responsible surgeon. Imaging, MRI of the local tumor, and chest CT are repeated 3 weeks after the last chemotherapy cycle. RECIST criteria will be used to evaluate clinical responses [5].

The surgery will be performed 4 to 6 weeks after the end of the third chemotherapy cycle, totaling 7 to 9 weeks after RT. If large dermal-adipose flaps must be fashioned, the aid of a skilled plastic surgeon can be requested to reduce wound morbidity. Figure 1 shows treatment schedule.

### Wound Complications

All cases are examined by a committee (including a nurse, surgeon, and oncologist) to evaluate and rate wound complications according to the criteria used by O’Sullivan et al in 2002; major wound complications were defined as a “secondary operation under general or regional anesthesia for wound repair or management” [8]. Late radiation toxicity grade will be reported according to the Radiation Therapy Oncology Group (RTOG-0630) [9].

### Pathological Anatomy

All cases are assessed by a single pathologist from the Department of Pathological Anatomy of AC Camargo Cancer Center (IWC) who will diagnose and grade the tumors per the World Health Organization Blue Books [2]. Tumor grades II and III are jointly considered to be high-grade for therapeutic purposes. The collection of clinical data and complementary exams will be obtained from medical records.

From each surgical specimen that is resected after preoperative chemotherapy treatment, a representative slice of the entire specimen with less visible macroscopic necrosis will be obtained. A mapping image will be generated from this slice with 1.0- to 2.0-cm^2^ cuts. Next, blocks and slides will be prepared for microscopic study. On microscopic examination, the percentage of viable cells, fibrosis, and necrosis in each slide will be determined. Ultimately, the sum of the percentages of viable cells in each slide will be divided by the number of slides evaluated. Based on this calculation, the mean value (as a percentage) of the viable cells that are present in the surgical specimen will be calculated.

### Statistical Analysis

SPSS version 18.0 (IBM Corp) will be used for all calculations. The variables and clinical outcomes of the study will be descriptive and will be determined based on simple frequencies of the variables themselves and the outcomes. McNemar tests will be used for paired categorical variables, and chi-square or Fisher exact tests will be used for unpaired categorical variables.

Measures of central tendency (mean and median) and variability (variance and standard deviation) will be used to describe numerical variables, whereas frequency distribution will be used for categorical variables. Mean and median follow-up times will be calculated from the date of the first cycle of chemotherapy until the date of the last consultation, loss to follow-up, or death. The level of significance for the statistical tests will be 5% (*P*<.05). Survival calculations and survival curves will be generated by the Kaplan-Meier method (Mantel-Cox) and log-rank test.

### Endpoints

The primary endpoints regard disease-specific survival and will be measured by the evaluation of local and distant disease-free survival after neoadjuvant treatment. Secondary endpoints are wound complication rates, amputation rates, and chemotherapy toxicity. We also will record the necrosis or viable cell rates after the schema and try to correlate with survival.

## Results

Patients have been recruited since February 2015, and recruitment is expected to be finished by the end of 2018. We are trying to change this protocol to a multicenter study in order to have 70 patients, based on the sample size calculation.

After the enrollment of the first 20 patients, an interim analysis is planned that focuses on side effects. The follow-up period will be at least 3 years after treatment, and it will include metastatic patients depending on the early results.

The main hypothesis is that the addition of hypofractionated RT can increase the necrosis rate, and we expect to have a higher rate of complete pathological response than in our previous 16% (data not shown), that was achieved using only chemotherapy. Whether this mechanism is related to survival remains unclear.

## Discussion

The prognosis of patients with high-grade STS remains poor, primarily due to relapses at distant sites [3]. Because STSs are rare, cases in most studies are separated into high-grade and low-grade tumors. This classification guides the therapy in most patients, although other factors such as tumor size and depth also correlate with relapse and death [1]. Tumor severity is another predictor of the response to cytotoxic therapies, wherein better response rates are observed with high-grade tumors. Thus, chemotherapy remains warranted for patients with high-grade tumors, irrespective of histological type, although some of them might respond differently to specific drugs [10,11].

Neoadjuvant chemotherapy,despite being a nonstandard treatment, can theoretically facilitate tumor resection with adequate margins and combat micrometastatic disease early and allow in vivo verification of the clinical or pathological response to chemotherapy after surgery. The pathological response that is induced by the treatment and the subsequent effects on survival have been the subjects of many studies [12-14], such as Gomez et al [15]. This group performed a retrospective study of patients with extremity high-grade sarcomas who underwent neoadjuvant treatment with RT and chemotherapy. Local disease-free survival and OS were significantly better in patients who exhibited more than 95% necrosis in the surgical specimen. The addition of ifosfamide to the regimen increased the percentage of patients with ≥95% necrosis from 13% to 48%, improving survival rates [16].

We used the standardized RECIST criteria [5] to evaluate the clinical response to preoperative treatment. The main limitation of this method is that it is one-dimensional with respect to the assessment of the primary lesion, because shrinkage of the tumor might be absent, but we believe that it is simple to implement and interpret. Consequently, the RECIST criteria remain widely used in measuring the clinical response to treatment for solid tumors [17].

Our previous results showed a wound complication rate (rWC) of nearly 42% using conventional chemotherapy/RT preoperatively, indicating that approximately 70% of patients failed to receive or were delayed in receiving adjuvant chemotherapy. Thus, in 2005, we began a new prospective phase 2 study using preoperative chemotherapy and relegating RT to the adjuvant scenario to reduce the surgical rWC. It was imperative to evaluate whether this preoperative chemotherapy regimen lowered the rWC without altering local control or amputation rates. Our group proposed that the action of systemic chemotherapy and its effect on the tumor, as a neoadjuvant treatment, be examined measuring the pathological response based on the quantification of necrosis, fibrosis, and the percentage of viable cells in surgical specimens.

From April 2005 to July 2012, 48 patients with high-grade extremity STSs were assessed; the median follow-up time was 40 months. The OS rates for nonmetastatic patients were 86.3% and 74.1% at 3 and 5 years, respectively, with an rWC of 19.5%. The analysis of surgical specimens showed that 6 patients (15%) had 5% (or less) viable cells in the sample, for whom the estimated OS and relapse-free survival (RFS) at 5 years were 100%. With a cutoff of 30% or less, 15 patients (37.5%) had an RFS of 82% and an OS of 90.9%.

Despite the favorable results with regard to avoiding the conventional preoperative RT scheme, the use of neoadjuvant chemotherapy alone has not effected better outcomes and subjects patients to higher doses due to larger fields of radiation postoperatively, correlating with late functional complications such as fibrosis and joint limitations. A different RT modality might solve or minimize this problem.

Hypofractionated treatment has comparable efficacy and safety as conventional fractionation in other solid tumors, such as those of the breast, prostate, and rectum. The usual dose for neoadjuvant RT in sarcomas is 50 Gy/25 fractions of 2 Gy. Radiobiology studies have shown that the sarcoma alpha/beta relationship is less than 10 Gy, which favors hypofractionated treatment [18]. Despite chemotherapy sometime being used concomitantly with RT, there is no standard treatment for hypofractionated RT. Ryan et al [19] prospectively treated 25 patients with hypofractionated RT (28 Gy/8 fractions) concomitant with chemotherapy with epirubicin and ifosfamide (3 cycles each pre- and postoperatively). This group reported high rates of hematological toxicity, with 64% of patients completing all chemotherapy cycles. Overall and disease-free survival was 84% and 62%, respectively.

More recently, a prospective study reported the results on 272 patients who were treated with an RT regimen that was similar to that in our study, with 25 Gy/5 fractions followed by immediate surgery (3 to 7 days after the end of RT) [20]. Some patients (22%) also underwent neoadjuvant chemotherapy. The OS rate at 3 years was 72%, with 42% of patients experiencing some acute toxicity and 7% requiring surgical intervention. The authors concluded that hypofractionated treatment generates similar results in terms of local control and OS compared with conventional treatment, with acceptable toxicity.

A criticism of chemotherapy for sarcomas is that histological types respond differently to several types of drugs. According to a multivariate analysis by the Italian and Spanish Sarcoma Group [21], histological subtype was associated with a difference in survival after chemotherapy, and patients with leiomyosarcoma had the worst prognosis. However, most studies claim that chemotherapy with doxorubicin must be maintained for most tumors of this type [22].

In this study, we added chemotherapy preoperatively to combat micrometastatic disease early and identify patients who were good responders (ie, harboring fewer than 30% viable cells in surgical specimens). For these responders who have received chemotherapy and hypofractionated RT, we offer 3 additional cycles of chemotherapy as an adjuvant treatment, avoiding chemotherapy for patients who theoretically do not need it.

Our main concerns are related to recruitment issues. Because STS is a rare condition, attaining the calculated sample size will be difficult. Most patients with high-grade STS are metastatic at the diagnosis, which is an exclusion criterion. However, as a cancer center, our institution receives patients from throughout Brazil, despite the lack of a specific government program.

Hypofractionated RT plus adjuvant chemotherapy can increase the pathological response and improve the outcomes of select patients, avoiding unnecessary chemotherapy and instead offering more chemotherapy to good responders. This approach is a rational use of adjuvant chemotherapy, despite the many histological types of sarcoma and limited chemotherapy schemes for sarcomas. We are confident that adding hypofractionated RT in the neoadjuvant scenario can shorten treatment times and lower costs without increasing the rWC compared with conventional RT.

## Abbreviations

CT: computed tomography
MRI: magnetic resonance imaging
OS: overall survival
RECIST: Response Evaluation Criteria in Solid Tumors
RFS: relapse-free survival
RT: radiotherapy
rWC: wound complication rate
STS: soft tissue sarcoma
